# Integrated palliative care in Europe: a qualitative systematic literature review of empirically-tested models in cancer and chronic disease

**DOI:** 10.1186/s12904-016-0130-7

**Published:** 2016-07-08

**Authors:** Naouma Siouta, K. Van Beek, M. E. van der Eerden, N. Preston, J. G. Hasselaar, S. Hughes, E. Garralda, C. Centeno, A. Csikos, M. Groot, L. Radbruch, S. Payne, J. Menten

**Affiliations:** Department of Radiation-Oncology and Palliative Medicine, University Hospital Gasthuisberg, Herestraat 49, 3000 Leuven, Belgium; Department of Anesthesiology, Pain and Palliative Medicine, Radboud University Nijmegen Medical Centre, Nijmegen, The Netherlands; International Observatory on End of Life Care, Division of Health Research, Lancaster University, Lancaster, UK; Department of Palliative Medicine, University of Navarra Hospital, Pamplona, Navarra Spain; Faculty of Medicine, Institute of Family Medicine, University of Pécs Medical School, Pécs, Hungary; Department of Palliative Medicine, University Hospital Bonn, Bonn, Germany

**Keywords:** Delivery of health care, Integrated, Palliative care, Review, Systematic, Medical oncology, Chronic disease

## Abstract

**Background:**

Integrated Palliative Care (PC) strategies are often implemented following models, namely standardized designs that provide frameworks for the organization of care for people with a progressive life-threatening illness and/or for their (in)formal caregivers. The aim of this qualitative systematic review is to identify empirically-evaluated models of PC in cancer and chronic disease in Europe. Further, develop a generic framework that will consist of the basis for the design of future models for integrated PC in Europe.

**Methods:**

Cochrane, PubMed, EMBASE, CINAHL, AMED, BNI, Web of Science, NHS Evidence. Five journals and references from included studies were hand-searched. Two reviewers screened the search results. Studies with adult patients with advanced cancer/chronic disease from 1995 to 2013 in Europe, in English, French, German, Dutch, Hungarian or Spanish were included. A narrative synthesis was used.

**Results:**

14 studies were included, 7 models for chronic disease, 4 for integrated care in oncology, 2 for both cancer and chronic disease and 2 for end-of-life pathways. The results show a strong agreement on the benefits of the involvement of a PC multidisciplinary team: better symptom control, less caregiver burden, improvement in continuity and coordination of care, fewer admissions, cost effectiveness and patients dying in their preferred place.

**Conclusion:**

Based on our findings, a generic framework for integrated PC in cancer and chronic disease is proposed. This framework fosters integration of PC in the disease trajectory concurrently with treatment and identifies the importance of employing a PC-trained multidisciplinary team with a threefold focus: treatment, consulting and training.

## Background

According to the World Health Organization (WHO), Palliative Care (PC) aims to improve the quality of life of patients and families who face life-threatening illness, by providing pain and symptom relief, spiritual and psychosocial support from diagnosis to end of life care and bereavement. Further, the WHO recommends that PC becomes an integral part of health care and that all patients affected by a life threatening disease should have access to PC services [1]. This statement is further supported by the European Association of Palliative Care [2] and is also in agreement with the guidelines of the European Council towards the European Union (EU) Member States [3].

This consensus in favour of integrating PC within regular treatment offered to patients with life-threatening disease is supported by a growing amount of evidence that indicates the effectiveness of PC on the improvement of the quality of life of these patients [4–8].

The implementation of integrated PC strategies is often based on models. Models of care are standardized designs that provide frameworks for the organization of care for people with a progressive life-threatening illness and/or for their (in)formal caregivers. As such, models determine the norms of PC practice and offer values and principles that professionals can use as guides and can thus provide important information for understanding integrated PC practices and evaluating their associated strengths and weaknesses.

To date, a univocally accepted model for PC delivery and integration does not exist, even if we confine ourselves to cancer or a major chronic disease. However, there are calls for developing generic PC models that will incorporate recent findings concerning the early identification of patients with needs for PC services and foster the integration of PC early in the care plan and throughout the disease trajectory [9–15].

In the present study, we perform a qualitative systematic review of the available literature for evidence-based models of integrated PC in Europe. We confine ourselves to studies that empirically measure the effectiveness of the corresponding models and employ a high-quality methodological rigour. Further, we examine the conformance of the included studies with respect to how the characteristics and requirements of integrated PC and strengths and weaknesses are documented. By incorporating the strengths and by rectifying the weaknesses, we propose a generic framework that aspires to demonstrate how to integrate PC both in cancer and chronic disease. This study is part of the European project InSup-C that focuses on integration of PC in advanced cancer and chronic disease in Europe (http://www.insup-c.eu/).

## Methods

A unanimously agreed definition of integrated PC does not exist as such. For the needs of the present study, a novel definition was developed in the course of the InSup-C meetings by the PC experts and the authors. This definition combines all the aspects of integrated PC, identified by both experts and literature. Our working definition is: “Integrated palliative care involves bringing together administrative, organisational, clinical and service aspects in order to realise continuity of care between all actors involved in the care network of patients receiving palliative care. It aims to achieve quality of life and a well-supported dying process for the patient and the family in collaboration with all the care givers (paid and unpaid)”.

It is important to note that this definition does not distinguish between timings that integration of PC can commence. Consequently, both early and end-of-life PC care can be part of this review.

Finally, this qualitative systematic review was conducted in Belgium in September of 2013.

### Selection criteria

Eligible studies included those focusing on models of integrated PC for adult patients with cancer or another chronic disease (COPD, renal failure, heart failure, HIV, dementia or other types of neurological diseases), that are, in turn, consistent with the above-mentioned definitions of models and of integrated PC. Since our primary objective concerns the identification of evidence based models, only those studies that empirically assessed the effectiveness of these models and provided relevant data were considered eligible. In particular, we considered randomized controlled trials (RCTs), quasi experimental studies, cohort studies, controlled before-and-after studies, observational studies and pilot evaluation studies whereas we excluded theoretical studies, audits, opinion-only studies in clinical case reports, editorials and letter.

We confided ourselves to studies published from 01-01-1995 (based on the publication year of the Calman-Hine report which constitutes the first national cancer plan in Europe [16]) to 31-12-2013 in one of the following languages: English, French, German, Dutch, Hungarian and Spanish which are the languages that the authors were knowledgeable of.

A final eligibility criterion concerned the quality of the included studies with respect to the methodological rigor. In the present systematic review, only studies that that scored at least 60 % (above 22/36) in the Hawker quality assessment scale were included in this systematic review; see Quality assessment section below.

### Search strategy

The following databases were searched electronically: The Cochrane Central Register of Controlled Trials (CENTRAL), PubMed, EMBASE, CINAHL, AMED, BNI, Web of Science and NHS Evidence. The search in the databases was performed via the use of keywords, MESH terms and search terms as well as their permutations and combinations. The basic search terms and keywords that were used in PubMed and similarly in the other electronic databases are presented in Appendix. Validation of the search strategy was performed against five key papers [4–8].

Additionally, the following journals were hand-searched: BMJ Supportive & Palliative Care, European Journal of Palliative Care, Journal of Pain and Symptom Management, Palliative Medicine and Medicina Paliativa. Citation tracking was also performed for the included studies.

Two different grey literature searches were performed. First, we identified and contacted experts in national scientific medical organizations in order to acquire additional information on existing models. Second, we carried out an electronic search in Google by utilizing a language-tailored strategy; more specifically, each country used relevant key-words and search terms translated into its corresponding language.

### Selection procedure

In the first phase, two reviewers (NS & KVB) screened all the search results on the basis of their title and their abstract. Non-English titles were screened and translated by two native speaker reviewers. The full texts of articles selected by both reviewers were sourced. Following standard practice, discrepancies were resolved by consensus.

### Data extraction

Data were extracted from papers meeting the inclusion criteria using an extraction form built upon the one described in Hawker et.al. This extraction form was modified for the purposes of this study following consensus in the InSup-C project meetings [17]. For each included paper, data extraction was carried out by the first two authors independently for the cross-checked their results and reached consensus on discrepancies. Extracted variables were: first author’s name, year of the study, country the study was conducted, design, quality assessment, description of the study model, outcome measures, results, focus of the model, setting, time frame of the model, disciplines represented and collaboration strategy.

### Quality assessment

The methodological rigour of each included study was formally assessed by the numerical scoring system designed by Hawker et al. [17]. This scoring system is based on nine criteria that are evaluated with a four-point Likert scale: good (4) to very poor (1), thus yielding a maximum score of 36. The nine criteria are: abstract/title, introduction/aims, methods/data, sampling, data analysis, ethics and bias, findings/results, transferability/generalizability, implications/usefulness. It is important to note that the criterion corresponding to methods takes into account the design of the study as well, e.g. RCT, observational study etc. It is on this premise that in the present systematic review we have included studies with different designs and not only RCTs. A detailed presentation of the Hawker tool is reported in Table 1. Our choice is also influenced by Oishi et al. and Rigby et al. who used the Hawker tool while conducting a PC-related systematic review [18, 19].

**Table 1:** Hawker tool description

1. Abstract and title: Did they provide a clear description of the study?	
Good	Structured abstract with full information and clear title.
Fair	Abstract with most of the information.
Poor	Inadequate abstract.
Very poor	No abstract.
2. Introduction and aims: Was there a good background and clear statement of the aims of the research?	
Good	Full but concise background to discussion/study containing up-todate literature review and highlighting gaps in knowledge.
	Clear statement of aim AND objectives including research questions.
Fair	Some background and literature review.
	Research questions outlined.
Poor	Some background but no aim/objectives/questions, OR
	Aims/objectives but inadequate background.
Very poor	No mention of aims/objectives.
	No background or literature review.
3. Method and data: Is the method appropriate and clearly explained?	
Good	Method is appropriate and described clearly (e.g., questionnaires included). Clear details of the data collection and recording.
Fair	Method appropriate, description could be better.
	Data described.
Poor	Questionable whether method is appropriate.
	Method described inadequately.
	Little description of data.
Very poor	No mention of method, AND/OR
	Method inappropriate, AND/OR
	No details of data.
4. Sampling: Was the sampling strategy appropriate to address the aims?	
Good	Details (age/gender/race/context) of who was studied and how they were recruited.
	Why this group was targeted.
	The sample size was justified for the study.
	Response rates shown and explained.
Fair	Sample size justified.
	Most information given, but some missing.
Poor	Sampling mentioned but few descriptive details.
Very poor	No details of sample.
5. Data analysis: Was the description of the data analysis sufficiently rigorous?	
Good	Clear description of how analysis was done.
	Qualitative studies: Description of how themes derived/respondent validation or triangulation.
	Quantitative studies: Reasons for tests selected hypothesis driven/numbers add up/statistical significance discussed.
Fair	Qualitative: Descriptive discussion of analysis.
	Quantitative.
Poor	Minimal details about analysis.
Very poor	No discussion of analysis.
6. Ethics and bias: Have ethical issues been addressed, and what has necessary ethical approval gained? Has the relationship between researchers and participants been adequately considered?	
Good	Ethics: Where necessary issues of confidentiality, sensitivity, and consent were addressed.
	Bias: Researcher was reflexive and/or aware of own bias.
Fair	Lip service was paid to above (i.e., these issues were acknowledged).
Poor	Brief mention of issues.
Very poor	No mention of issues.
7. Results: Is there a clear statement of the findings?	
Good	Findings explicit, easy to understand, and in logical progression.
	Tables, if present, are explained in text.
	Results relate directly to aims.
	Sufficient data are presented to support findings.
Fair	Findings mentioned but more explanation could be given.
	Data presented relate directly to results.
Poor	Findings presented haphazardly, not explained, and do not progress logically from results.
Very poor	Findings not mentioned or do not relate to aims.
8. Transferability or generalizability: Are the findings of this study transferable (generalizable) to a wider population?	
Good	Context and setting of the study is described sufficiently to allow comparison with other contexts and settings, plus high score in Question 4 (sampling).
Fair	Some context and setting described, but more needed to replicate or compare the study with others, PLUS fair score or higher in Question 4.
Poor	Minimal description of context/setting.
Very poor	No description of context/setting.
9. Implications and usefulness: How important are these findings to policy and practice?	
Good	Contributes something new and/or different in terms of understanding/insight or perspective.
	Suggests ideas for further research.
	Suggests implications for policy and/or practice.
Fair	Two of the above (state what is missing in comments).
Poor	Only one of the above.
Very poor	None of the above.

### Data synthesis

The included studies were characterized by a substantial heterogeneity as studies with both clinical and methodological diversities were included. For this reason, a narrative synthesis was favoured over a meta-analysis and data are reported in tables; hence the qualitative nature of the review mentioned above. This heterogeneity has also affected the presentation of the results. Comparative analysis was only possible by using a specific number of categories (design, study populations, assessments and outcomes) that will be presented in the following sections.

## Results

**Fig. 1:**
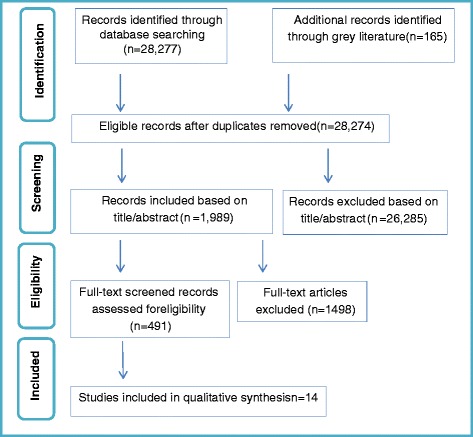
Flow diagram of study selection procedure

The database search returned 28,274 hits, excluding duplicates. From the studies that were screened based on abstract/title, 1989 were found to be eligible for full-text screening. Upon full text screening, 491 studies qualified for further assessment of their eligibility. Finally, 14 studies were found to comply with all the selection criteria (see relevant section above). The grey literature search did not add any empirical studies. A flow diagram of the selection procedure and the results is shown in Fig. 1.

From the 14 studies, 7 evaluated models about chronic disease (dementia, multiple sclerosis, chronic heart failure, HIV/AIDS and advanced chronic disease in general), 4 about integrated care in oncology, 3 about cancer and chronic disease (end-of-life patients). The majority of the included studies were from the UK (6 out of 14), 2 were from Spain, 2 from The Netherlands, and one each from Italy, Germany, Norway and France. The preponderance of UK studies is clear in chronic disease where all 6 included studies were from this nation.

The 14 included studies were built upon quite different designs (Table 2) [20–33]. More specifically, there were 6 RCTs, 2 observational studies, 2 cohort studies, 2 pilot evaluation studies, 1 uncontrolled before-and-after studies and 1 quasi-experimental study.

**Table 2:** Characteristics of the included studies according to different disease categories

First Author, Year, Country	Design	Quality Assessment	Model	Outcome measurements	Results/Effectiveness of the model
Cancer					
Jordhøy et al. 2001, (Norway) [20]	Cluster Randomised Trial	33	Collaboration between palliative medicine unit and community service	HRQL*: physical, emotional functioning, pain, psychological distress. Place of death, hospital utilisation.	There was no evidence of any impact on the patients’ HRQL*. There was no tendency in favour of any treatment group on the main outcomes in assessments that were made within 3 months before death.
Smeenk et al. 1998, (The Netherlands) [21]	Quasi-experimental study	31	Transmural home care programme: collaboration primary care team and supporting hospital care team.	Re-hospitalization, QoL*, home death	Patients in the intervention group underwent significantly less re-hospitalization during the terminal phase of their illness; the intervention contributed significantly positive to the patients physical QoL; A higher, not significant percentage died at home.
Colombet et al. 2012, (France) [22]	Case series study nested in a cohort	30	Impact of oncologist’s awareness of PC, clinical intervention of PCT* and timing, multidisciplinary decision-making.	Indicators: location of death, number of ER* visits in last month of life, chemotherapy administration in last 14 days of life.	58 patients died at home, 45 in an ICU or ER, and 253 in an acute care hospital; 185 patients visited the ER* in last month of life and 75 received chemotherapy in last 14 days of life. OPM* independently decreases the odds of receiving chemotherapy in last 14 days of life. Early PCT* intervention had no impact on indicators, whereas OPM* reduced the odds of persistent chemotherapy in the last 14 days of life. Decision-making with oncologists and the PC* team is the most critical parameter for improving EoLC*.
Schreml et al. 2000, (Germany) [23]	Observational study	25	Integration of PC into regular internal ward services in general hospital.	Release of pain, respiratory distress, dying in hospital, length of hospital stay.	It is possible to integrate PC into a regular internal medical ward, with positive impact on recorded outcome measures.
End-of-life (Liverpool Care Pathway)					
Constantini et al. 2013, (Italy) [24]	Cluster Randomised Trial	34	LCP* for cancer patients dying in hospital medical wards.	Quality of EoLC* from perspective of bereaved family member; communication between ward staff and GPs* (VOICES)*.	Aspects of quality of EoLC* improved (emotional, spiritual needs, self-efficacy); slight improvement in communication, no significant improvement in symptom control.
Veerbeek et al. 2008, (The Netherlands) [25]	Uncontrolled before and after study	27	Effect of the LCP* on 3 health care settings (hospital, nursing home, home).	Comparison of level of documentation, symptom burden (EORTC QLQ-C30* questionnaire) and aspects of communication (VOICES)* before and after introduction of LCP*.	Introduction of LCP* increased documentation, decreased symptom burden.
Malignant and Non-malignant Disease					
Grande et al. 2000, (UK) [26]	Randomised controlled trial	33	Cambridge Hospital at Home for palliative care (CHAH).	Symptom control, adequacy of care, likelihood of remaining at home in their final 2 weeks, GP visits.	CHAH appeared to be associated with better quality home care.
Vicente et al. 2010, (Spain) [27]	Retrospective and prospective cohort study	30	Influence of the Integrated Plan of PC* of the Autonomous Community of Madrid in the medical activity of a hospital based PC* unit.	Improvement in continuity of care, coordination amongst assistant bodies, increase in mean stay at the PCU*, increase in number of home deaths, etc.	PC home care improves continuity in care of patients. Transfers to intermediate stay care centers and deaths at home increased. Median stay at the PCU* decreased.
Dementia					
Sampson et al. 2011, (UK) [28]	Randomised controlled trial	28	Pilot implementation of the assessment of PC needs of patients with severe dementia and discussion with principal carers to improve EoLC*.	Kessler Distress Scale, EQ-5D*, Decision Conflicts Scale, Decision Satisfaction Inventory, State Anger Scale, Life Satisfaction Scale, Satisfaction with EoLC*, Advanced Dementia Scale (FAST Scale); Pain and distress (the Abbey pain scale, the PACSLAC and the Doloplus), delirium (Confussion Assessment Method),	General unwillingness to address EoL* issues. All carers were keen to receive more information about EoL* issues in dementia, found discussions very helpful. Participation of clinical MD* team facilitated integration of intervention with the clinical service.
Multiple Scherosis					
Higginson et al. 2009, (UK) [29]	Randomised controlled trial	36	Evaluation of cost-effectiveness of a new PC service for people with MS*.	Use of services, patient symptoms (UNDS*, EDSS* and POS-8*), other outcomes, caregiver burden (ZBI*).	Short-term PCT* was found to be cost-effective, reducing inpatient and community costs, caregiver burden and possibly patient pain.
Edmonds et al. 2010, (UK) [30]	Randomised controlled trial	36	Evaluation of a novel PC service.	MS Impact Scale, POS-8*, ZBI*, Modified Lawton positivity questionnaire.	MS patients who received PC* service had improvements in 5 key symptoms (pain, nausea, vomiting, mouth problems and sleeping difficulties) on the POS and improved informal caregiver wellbeing.
HIV/AIDS					
Koffman et al. 1996, (UK) [31]	A descriptive pilot evaluation	23	Pilot evaluation of hospice at home service for patients with advanced HIV/AIDS; 24-h terminal care.	STAS*, evaluating pain control, other symptom control, patient/family anxiety, patient/family insight and communication between patient and family, between professionals, between professionals and patient and family.	80 % died at home; STAS* showed improvements in items ‘other symptoms control’ and family insight.
Chronic Heart Failure					
Pattenden et al. 2012, (UK) [32]	Non-randomised pilot evaluation	30	Collaborative PC* for advanced heart failure.	Death in preferred place of care; hospital admissions averted; costs of medical procedures, inpatient care and directs costs of intervention.	This pilot study provides tentative evidence that a collaborative home-based PC* service for patients with advanced CHF may increase the likelihood of death in place of choice and reduce inpatient admissions.
Advanced Chronic Disease					
Navarro et al., 2011, (Spain) [33]	Observational, retrospective and descriptive study	26	EoLC* of advanced chronic non-cancer patients identified by multidimensional evaluation and interdisciplinary teamwork in a medium and long term hospital.	General data, terminal criteria, diagnostic and prognostic information, development of advance directives, limiting levels of effort care, times from admission, risk of complicated bereavement.	Identification of advanced chronic non-cancer patients and their needs by interdisciplinary teamwork enabled indication for PC soon after admission and ensured appropriate care during their stay.

Table 2 describes studies by each disease category according to author, year, country, design, quality assessment score, model, outcome measures and results/findings

*Abbreviations: PC* palliative care, *QoL* quality of life, *PCT* palliative care team, *OPM* onco-palliative meeting, *EoL* end of life, *VOICES* Views of Informal Carers – Evaluation of Services questionnaire, *ER* emergency room, *MDT* multidisciplinary team, *QoC* quality of care, *GSFCH* Gold Standards Framework in Care Homes, *LCP* Liverpool Care Pathway, *PCU* palliative care unit, *UNDS* United Kingdom Neurological Disability Scale, *EDSS* Expanded Disability Status Scale, *POS-8* Palliative Care Outcome Scale, *ZBI* Zarit Carer Burden Inventory, *ESH* Hospital Support Team, *EQ-5D* EuroQOL five dimensions questionnaire, *EoLC* end of life care, *MD* medical doctor, *MS* multiple sclerosis, *STAS* Support Team Assessment Schedule, *ICU* intensive care unit, *CKD* Chronic Kidney Disease

Table 2 provides information concerning the quality of the included studies, as measured via the Hawker et al. tool. Two (2) studies [29, 30] scored a perfect 36 while 3 other studies scored 33 or 34. The majority of the remaining studies scored in the range between 23 and 31. Table 2 includes detailed information concerning the models of the included studies, their outcome measures and the results/effectiveness of the corresponding models. From this table, we can infer that although RCTs tend to score higher and observational studies lower, other designs perform reasonably well. The latter indicates that such studies have a sound basis, despite the fact that they do not employ an RCT for their assessment.

Even though some of the models bear similarities, (see Table 3 Screening), differences associated with the care setting and the compositions of the team render further grouping and comparison difficult. Upon detailed examination, a common ground for comparison was found in the following categories: focus of intervention, the setting, the timing of the intervention in the disease trajectory, the collaboration strategy and the effectiveness of the models. In the following section, we confine the presentation of the results to these categories.

**Table 3:** Characteristics of the models

First author	Focus of the model	Setting	Time frame	Disciplines represented	Collaboration strategy
Cancer					
Jordhoy et al. [20]	treating, training, consulting	Hospital, GP’s, nursing homes, home care	end of life	GP, community nurse, consultant nurse, physician from PMU.	Model-responsible team meetings^a^
Smeenk et al. [21]	treating, training, consulting	Hospital, primary care team	concurrent, end of life	Specialist nurse coordinator, oncology ward nurses + medical specialist, transmural home team with nurses from hospital + day care.	Model-responsible team meetings, protocol
Colombet et al. [22]	treating, consulting	Hospital	concurrent, end of life	15 referral physicians: oncologists who prescribe chemotherapy, of whom 2 have been trained in PC fundamentals. MDT: PCT and oncology staff. PCT: PC specialists, nurses, secretary assistant, psychologist. Oncology staff: physicians, nurses, head nurses, social workers, psychologists, secretaries.	Model-responsible team + additional experts meeting^b^
Schreml, et al. [23]	treating, training, consulting	Hospital	concurrent, end of life	Physicians and nurses.	Model-responsible team meetings
End-of-life (Liverpool Care Pathway)					
Constantini et al. [24]	treating, training	Hospital	end of life	PCT: 2 physicians, 3 nurses, 2 psychologists.	protocol
				LCP training: nurses and physicians of the hospital wards.	
Veerbeek et al. [25]	treating	Hospital, nursing home and home	end of life	Physicians and nurses.	Model-responsible team meetings, protocol
Malignant and Non-malignant Disease					
Grande et al. [26]	treating	Home	end of life	Six qualified nurses, 2 nursing auxiliaries, CHAH coordinator, agency nursing care.	Model-responsible team meetings
Vicente et al.[27]	treating	Hospital, home	end of life	PC home team as an MDT comprised by physicians, nurses, nurse assistants, and administrative assistants, social workes, psychologists.	Model-responsible team meetings
Dementia					
Sampson et al. [28]	treating, training, consulting	Hospital, home	end of life	Senior nurse experienced in dementia and trained in PC; clinical MDT.	Model-responsible team meetings
Multiple Sclerosis					
Higginson et al. [29]	treating, consulting	Home, hospital outpatient clinic, care homes, hospital	concurrent	Part-time PC medicine consultant, 1 part-time clinical nurse specialist, 1 administrator, 1 psychosocial worker.	Model-responsible team meetings
Edmonds et al. [30]	treating, consulting	Home, hospital outpatient clinics, care homes, hospital	concurrent	Part-time consultant in PC Medicine with specialist interest in neurological conditions, part-time clinical nurse specialist, full time administrator.	Model-responsible team + additional experts meeting
HIV/AIDS					
Koffman et al. [31]	treating, consulting	Hospice, home	end of life	Nurses trained in PC; bank nurses for night-sitting; 2 PC medicine consultants.	Model-responsible team + additional experts meeting
Chronic Heart Failure					
Pattenden et al. [32]	treating, consulting	Homes, hospice, ‘care of the elderly’ wards	concurrent, end of life	Heart Failure nurse specialists, MCN nurses, MCN health care assistants, cardiology, ‘care for the elderly’ consultants, district nurses, GPs.	Model-responsible team + additional experts meeting, protocol
Advanced Chronic Disease					
Navarro et al. [33]	treating	Hospital	concurrent	The MDT consists of physicians, head nurse, ward nurses, auxiliary nurses, collaborating with a dietician, psychologist, social worker, rehabilitation physician, physiotherapist, occupational therapist and speech therapist.	Model-responsible team + additional experts meeting

Table 3 describes five characteristics of the included studies: the focus of the model, the setting, the time frame of the model, the functions represented, and collaboration strategy involved

*PC* palliative care, *GP* general practitioner, *PMU* palliative medicine unit, *PCT* palliative care team, *MDT* multidisciplinary team, *LCP* Liverpool Care Pathway, *MNC* Marie Curie Cancer Care, *CHAH* Cambridge hospital at home service, *ESAD* Home Care Support Team, *MDM* multidisciplinary meetings

^a^meetings of the team that is involved in the implementation of the model

^b^meetings between the team responsible for the implementation of the model and other disciplines involved in the treatment of the patient

### Focus of intervention

All the included studies placed their focus on symptom treatment. In particular, the objective of these interventions was to improve physical symptoms (dyspnoea, pain, constipation, nausea, vomiting, diarrhoea), emotional symptoms (agitation, confusion, fear, delirium) and to a lesser extent cognitive and social functioning [20, 23, 28–31]. Nine studies focused on consulting about end-of-life care decisions, advance care planning, advice for coping with life-threatening disease and referrals to specific care-settings. Five studies focused on the training of the nurses and physicians involved in the interventions. The various training programmes included training of the staff to support patients over the telephone [21], education on the symptoms of the disease [28] and more general educational programmes for community professionals about palliative care services [20, 24, 28].

### Setting

The implementation of the models has taken place in a variety of settings. In fact, most studies took place in more than one setting with the majority focusing on inpatient (11/14) and home care (9/11).

### Timing of PC initiation

As regards the timing of the iniation of PC, three types of studies were identified; those focusing on end-of-life (11), on concurrent care setting (where PC care is provided alongside regular treatment)(3) and on both (4). For the end-of-life setting, included patients typically were expected to die within a range spanning from a few weeks (2 weeks [26]) to few months (e.g. 2–9 months [20]). For the concurrent care setting, eligible patients were referred by clinicians as potentially benefiting from PC assessments [29, 30]. Finally, for the combined setting, various eligibility categories were identified. For example, in [23] all patients with malignant disease independent of the disease trajectory were eligible.

### Composition of team

Three different team compositions were identified from the included studies. The first type refers to teams comprising medical and nursing staff; 2 studies [23, 25]. The second type concerns multidisciplinary teams comprising different professions: general practitioners (GPs), medical specialists, nurses and specialist nurses, psychiatrist and psychologists, health coordinators, administrative assistants, social workers, ambulance services, dieticians and rarely occupational therapists and speech therapists; 4 studies [21, 26, 33]. The third type corresponds to multidisciplinary teams that additionally involve PC experts. Besides being multidisciplinary, these teams also have PC physicians, PC nurses and PC psychologists; 8 studies [22, 24, 31].

### Collaboration strategy

The collaboration strategy refers to the ways that the represented disciplines cooperate and assess emerging issues. As shown in Table 3, the included studies employ three different collaboration strategies and their combinations: i) meetings of the team that is involved in the implementation of the model (model-responsible team) [20, 21, 23–28], ii) meetings between the model-responsible team together with experts from other disciplines involved in the treatment of the patient [22, 29, 31–33], iii) utilization of predefined protocols [21, 24, 25, 32]. It is important to note that the collaboration is not necessarily related to the composition of the team. For example, a model-responsible team may be multi-disciplinary but still base its decisions on meetings where additional disciplines are represented. In other words, the principal difference between (i) and (ii) is the participation of experts that are not directly related to the implementation of the model in question on a frequent basis.

### Effectiveness of the model

As regards effectiveness, all but one study [20], showed positive outcomes in terms of better symptom control and better quality of life, better communication between personnel, patients and caregiver, more deaths at the patients’ homes and more cost-effective care. However, this result should be interpreted with care given the variability in the design of the interventions, some of which were not RCTs and therefore of lower quality.

Importantly, even within RCTs, that constitute the most robust design, further comparisons between the results are cumbersome to perform. This is because discrepancies are observed both in the outcome measures and in the tools employed for their assessment. For example, as regards the evaluation of symptom management, some of the studies used the European Organization for Research and Treatment of Cancer guidelines (EORTC QLQ-C30) questionnaire [24, 25, 34], whereas others employed the United Kingdom Neurological Disability Scale (UNDS) [35], the Expanded Disability Status Scale (EDSS) [36], the Palliative Care Outcome Scale (POS-8) [37] and the Support Team Assessment Schedule (STAS) [29–31, 38] or the Abbey pain scale [39], the PACSLAC [40] and the Doloplus [28, 41]. Also, communication aspects were assessed via the Views of Informal Carers-Evaluation of Services (VOICES) [42] tool [24, 25]. Further, caregivers’ burden was measured with the help of the Zarit Carer Burden Inventory (ZBI) [43], [29, 30]. Finally, stressful events were assessed via the Impact of Event Scale (IES) questionnaire [44].

## Discussion

We reviewed existing literature on evidence-based models for integrated PC in patients with cancer or chronic disease in Europe. Since the focus of the present study was on the effectiveness of existing models, only specific designs were considered as eligible e.g. RCTs, cohort studies, etc. whereas others, such as theoretical studies, audits, etc., were explicitly excluded. In addition, in order to exclude empirical studies that were conducted with low-quality standards, the numerical tool of Hawker et al. [17] was employed for the assessment of the methodological rigour. In this respect, only those studies that scored at least 60 % in this tool were eligible.

The database search resulted in 14 empirical studies that fulfilled the inclusion criteria. According to our findings, there are only a few models of integrated PC in Europe whilst the geographic distribution of the origin of these interventions is imbalanced, with 6 out of 14 studies coming from the UK.

Only 6 out the 14 studies were RCTs whilst the remaining employed different designs, e.g. observational studies. However, as mentioned above, studies employing a different design for their assessment scored highly on Hawker’s tool as well. Consequently, such non-RCT studies can provide valuable insight into the strengths and weaknesses of extant studies that has to be taken into consideration if a complete description of the current state-of-the-art is targeted, as is the case of the present study.

Even though the included studies employ different outcome measures and a direct comparison is not possible, the effectiveness of the models (as assessed by the outcome measures) was confirmed in 13 out of 14 studies. In particular, as mentioned above, the positive impact typically involves better symptom control, higher quality of life, less caregiver burden, improvement in continuity and coordination of care, fewer hospital admissions, patients dying more frequently at home, and improved cost-effectiveness. Even though only 6 out of 14 studies were RCTs, and thus the results of the assessments for the remaining studies should be treated with care, the positive outcomes still add to the growing bulk of evidence corroborating the effectiveness of both usual and integrated PC on the improvement of the quality of life of patients with life-threatening disease [4–8].

We now shift to the development of a framework of integrated PC that could serve as a first step towards the development of a generic model for both malignant and non-malignant disease. Although the included studies suggest practices that are in concordance with existing recommendations and empirical findings, no study presents an intervention that is optimal in all aspects. Consequently, the generic framework that we propose is based on a selective combination of the strengths of the included interventions alongside a critical evaluation of their weaknesses and shortcomings. First, we present the aspects of the generic framework and subsequently we rationalize our choice and motivations.

This generic framework consists of the following aspects.

- Focus of intervention: The focus of intervention will be placed on symptom treatment, consulting of patients/family and training of the personnel.
- Setting: The design of the framework is such that it can be applied to every care setting.
- Timing of intervention: The intervention can be initiated throughout the disease trajectory either concurrently or in the end-of-life.
- Composition of team: The framework requires a multidisciplinary team with members that are trained in the delivery of PC. This team can consist of GPs, physician specialists, nurses and specialist nurses, psychologists, social workers and administrative assistants.
- Collaboration strategy: The collaboration strategy, which refers to the ways that the represented disciplines cooperate and assess emerging issues, should be based on the involvement of the multidisciplinary team and its meetings and the utilization of protocols.

The proposed framework has a threefold focus: treatment, consulting and training. In our context of integrated PC, treatment corresponds to the alleviation of the physical (dyspnoea, pain, constipation, nausea, vomiting, diarrhoea) and psychological symptoms (agitation, confusion, fear, delirium). On the other hand, consulting refers to discussions and communications over treatment options and at a later stage to end-of-life care decisions. Moreover wishes, desires and needs of the patients can be taken into account as part of this advance care planning. Training involves the education of nurses, physicians and other related health care providers involved in the interventions. Training programmes can be very diverse, however, their principal focus should be on PC, its benefits and its provision.

The benefits of focusing on treatment are apparent, however, the benefits of the remaining two aspects are also well documented. As regards consulting, PC typically involves difficult medical and ethical decision making [4–8] for patients and their (in)formal caregivers. Importantly, the same studies assert that the burden associated with this decision making can be ameliorated with the assistance of trained PC staff through systematic consulting and informative discussions.

By contrast, regarding training, we first note that the generic framework should be utilized with extreme caution because these suggestions may need to be tailored to the specific situation. In other words, this framework pertains to assist PC teams in their planning and implementation of PC strategies and not to provide black-box solutions. In this respect, it should be highlighted that the improper application of generic frameworks, and in fact of every integrated PC framework, can jeopardize the effectiveness of the employed PC practices. A notable example is the case of the Liverpool Care Pathway (LCP) [45], which has been sometimes erroneously employed, leading to poor outcomes and has in turn been used as an excuse for poor quality care [46, 47]. Public outcry from the poor implementation of the LCP led the UK government to stop the use of the LCP.

Recent studies explicitly state that there is a correlation between the successful implementation of the LCP and the training of the staff [48]. Importantly, this constitutes a characteristic of integrated PC in general [49–51]. For this reason, future efforts should explicitly foster the educational aspect of the professional members.

The choice of the timing of the intervention and thus the referral criteria is a contentious topic in the literature of PC. Historically, PC studies focusing on cancer opted for an end-of-life time frame based on the premise that once cancer reaches the terminal phase, the disease trajectory declines rapidly so that end-of-life prognoses can be fairly accurate [52, 53]; still, prognosis in cancer is frequently over-optimistic [54]. However, early integrated PC is increasingly supported by various studies, because it has been documented to improve quality of life in the last days and increase survival [4].

Moreover, end-of-life prognosis for non-malignant disease is problematic. For example, the typical trajectory of Chronic Heart Failure (CHF) consists of acute crises or exacerbations followed by elongated periods of stability. At the same time, CHF patients are in high risk of sudden death [55]. Consequently, for non-malignant disease, an end-of-life frame is cumbersome to realise and a concurrent time frame of the initiation of PC is deemed more appropriate [56]. On the other hand, there is a growing number of studies supporting the decoupling of the initiation of PC from the traditional end-of-life time frame and the shift to the early integration of PC in the disease trajectory [57–61].

According to our definition of integrated PC, the timing of the initiation of PC care is not determined so that models that focused on either concurrent PC or end-of-life care were eligible. Despite the fact that, as mentioned above, end-of-life care in patients with non-malignant disease is difficult to realise in practice, this is not reflected in our results since representatives of both categories are included for patients with either malignant or non-malignant disease. As a consequence, our generic framework necessarily employs a diverse number of timings, ranging from concurrent to end-of-life. This is not without implications as offering a spectrum of options, over a uniquely defined one, implies that the final choice falls within the jurisdiction of the involved teams. In turn, this might lead to significantly diverse practices and, therefore, impede the benefits associated with PC. Future studies should thus focus on the convergence of opinions on the timing of PC so that ideally a standardized recommendation is crafted.

In the proposed generic framework, the involved team should be multidisciplinary in nature and involve experts from different fields. Functionality of multidisciplinary teams may be impeded by ambiguity of the distributed roles, conflicts between the team members, communication problems and issues related to leadership [62, 63]. However, extant studies in PC for patients with both malignant and non-malignant disease support the results of this study showing that multidisciplinary teams (MDTs) lead to better results when compared to uni-disciplinary ones because they enhance continuity of care, evidence-based decision making, ACP and high quality care delivery [4, 64–66].

As the integration of PC requires bringing together specialists from different backgrounds and their well-orchestrated coordination, the development of protocols that quantify their collaboration is imperative for the successful implementation of PC. As mentioned in the Results section, the included studies employ miscellaneous collaboration strategies consisting of distinct components: collaboration between the team members (intra-team collaboration), multidisciplinary team meetings and protocols. The intra-team collaboration, which constitutes the only common component of all studies, is important because it assures the efficient implementation of the PC strategy. Further, for the aforementioned reasons multidisciplinary team meetings are also important for enhancing the continuity of care. Lastly, protocols have compelling advantages because they provide a clear guide for the next steps to be undertaken [67]. Since none of these components are redundant and our results show that they are complementary to each other in our generic framework, we propose to employ a strategy based on a combination of these.

Having addressed all the components of this generic framework, a comment on the implications that this framework has on the recommendations of the WHO [1] is in order. Overall, the proposed framework accords very well with the definition of PC provided by the WHO, however, two important differences are identified. First, the proposed framework highlights the training of the healthcare providers, which is essential for the successful implementation of integrated PC; this is not the case of the WHO definition where training is absent. Second, although WHO recognizes the need for employing a team approach no explicit reference on multidisciplinarity is made. By contrast, the proposed framework emphasizes the importance of utilizing PC teams the members of which come from various backgrounds.

Finally, it is interesting to juxtapose the timings of PC. As mentioned above, due to the fact that the InSup-C definition of integrated PC does not comment on the timing, the proposed framework offers a multitude of timings; from concurrent to end-of-life. The definition of WHO states that PC “is applicable early in the course of illness, in conjunction with other therapies that are intended to prolong life…”. However, as stated, this phrase does not explicitly promote early integration but rather on its range of applicability. In other words, the WHO definition does not narrow down the timing of PC to concurrent but rather expends to a spectrum of option in a manner similar to the proposed framework. Consequently, if attention has to be shifted to a concurrent timing, this should be explicitly reflected in the corresponding definitions.

Additional information may be drawn by comparing the five aspects of the generic framework against indicators for integrated PC. Such sets of indicators do exist in the literature, however, they correspond to PC in patients with cancer. Still, since cancer also pertains to the present such a comparison is fruitful. A recent systematic review provided a set of 38 indicators for integrated PC in oncology [68]. These indicators were grouped into five groups namely: structure of clinical programs, processes of clinical programs, education, research and administration. The same grouping was employed in the subsequent study [68], where a set of 43 indicators (13 major and 30 minor) was proposed based on a consensus of experts. Interestingly, with the exception of research, the generic framework, and the models that has been composed from, satisfies the majority of these indicators for both studies.

### Study limitations

The first limitation of this study concerns the broadness of the topic under investigation. Integrated PC in cancer and non-cancer patients and without time specification is a very large area and requires a quite generic search strategy. Moreover, in view of this broadness, we were confronted with a substantially heterogeneous set of results that could only be accessed through a qualitative description.

The search strategy employed was developed upon consensus between the PC experts involved in the project meetings. However, a unanimously agreed definition for integrated PC does not currently exist. Consequently, even though our strategy is broad, some evidence might have been missed due to the keywords used for the search strategy.

A third limitation concerns the choice of the languages of the included studies. The present study is confined to studies published in Dutch, English, French, German, Hungarian and Spanish, that were the languages that the project partners were knowledgeable of. It is, therefore, possible that studies published in other EU languages are available and not included [69].

Finally, we stress that the generic framework proposed herein has been based on models for patients with cancer and non-cancer disease. Its validity, therefore, is formally limited to these particular diseases. Although extrapolations to other types of life-threatening disease are tempting, further study in this direction is clearly needed. Also even though the proposed framework aims to be generic, one has to acknowledge limitations stemming from differences between healthcare systems, cultural and geographic factors and funding levels. Moreover, the preponderance of UK studies in this review should not go unnoticed. This is further supported by [70] who argue that generic models have to be flexible enough to be tailored to different settings; as a consequence, their recommendations should not be extremely stringent.

## Conclusions

This is the first qualitative systematic literature review of models on integrated Palliative Care (PC) in patients with malignant and non-malignant disease in Europe. It represents the combined effort of an international consortium of six partners in six different European countries (Belgium, Germany, Hungary, Spain, The Netherlands and United Kingdom).

Based on the results, we have proposed a generic framework for integrated PC in patients with cancer and chronic disease. The proposed generic identifies the importance of employing a PC-trained multidisciplinary team and of having a threefold focus: on treatment, consulting and training. Each component of our framework has been already empirically assessed, however, the overall framework has not. Although one could extrapolate the existing empirical evidence in favour of our framework’s efficacy, we believe that an empirical study for the framework as a whole should carried. Such a study not only will provide empirical evidence on the framework’s efficacy but will also shed light on possible weaknesses that can be only traced post-implementation. Additionally, it would be interesting to conduct the same systematic review in non-European countries and performed a comparative analysis in a worldwide scale.

## Appendix

In this appendix we present the search terms that were used for our search strategy.

*“(hospices OR supportive care OR supportive care* OR end of life care* OR palliative* OR palliative care [MeSH Terms] OR hospice* OR terminal care* OR coordinated care* OR integrated care* OR transmural care* OR progressive patient care*) AND (“end stage disease” OR end stage disease* OR dying OR death [MeSH Terms] OR Chronic disease [MeSH Terms] OR Chronic disease* OR terminally ill* OR terminally ill [MeSH Terms] OR cancer) AND (care pathway* OR care pathway OR pathway* OR patient transfer* OR patient transfer OR patient care team* OR managed care program* OR continuity of patient care OR patient care management OR patient care plan* OR patient care planning OR illness trajectory OR “advance care planning” OR advance care planning OR delivery of health care OR models of care OR model of care OR model organizational OR models organizational OR organizational model* OR guideline*) NOT ((birth) OR child) OR pediatrics)) NOT ((animals[mh] NOT humans[mh])) Filters: Publication date from 1995/01/01 to 2013/12/31”.*
